# Vitamins A & D Inhibit the Growth of Mycobacteria in Radiometric Culture

**DOI:** 10.1371/journal.pone.0029631

**Published:** 2012-01-03

**Authors:** Robert J. Greenstein, Liya Su, Sheldon T. Brown

**Affiliations:** 1 Department of Surgery, James J. Peters Veterans Affairs Medical Center, Bronx, New York, United States of America; 2 Laboratory of Molecular Surgical Research, James J. Peters Veterans Affairs Medical Center, Bronx, New York, United States of America; 3 Department of Medicine, James J. Peters Veterans Affairs Medical Center, Bronx, New York, United States of America; 4 Mount Sinai School of Medicine, New York, New York, United States of America; Universita di Sassari, Italy

## Abstract

**Background:**

The role of vitamins in the combat of disease is usually conceptualized as acting by modulating the immune response of an infected, eukaryotic host. We hypothesized that some vitamins may directly influence the growth of prokaryotes, particularly mycobacteria.

**Methods:**

The effect of four fat-soluble vitamins was studied in radiometric Bactec® culture. The vitamins were A (including a precursor and three metabolites,) D, E and K. We evaluated eight strains of three mycobacterial species (four of *M. avium* subspecies *paratuberculosis* (MAP), two of *M. avium* and two of *M. tb.* complex).

**Principal Findings:**

Vitamins A and D cause dose-dependent inhibition of all three mycobacterial species studied. Vitamin A is consistently more inhibitory than vitamin D. The vitamin A precursor, β-carotene, is not inhibitory, whereas three vitamin A metabolites cause inhibition. Vitamin K has no effect. Vitamin E causes negligible inhibition in a single strain.

**Significance:**

We show that vitamin A, its metabolites Retinyl acetate, Retinoic acid and 13-cis Retinoic acid and vitamin D directly inhibit mycobacterial growth in culture. These data are compatible with the hypothesis that complementing the immune response of multicellular organisms, vitamins A and D may have heretofore unproven, unrecognized, independent and probable synergistic, direct antimycobacterial inhibitory activity.

## Introduction

Since early in the last century [1] the role of both vitamin A (see [2] for review) and vitamin D (see [3], [4] for review) in combating infectious diseases has been investigated. It is noteworthy that in the vast majority of studies, the underlying assumption has been that any efficacy of these vitamins in combating disease is consequent to enhancement of the immune response of the infected host [5]–[8]. There is no direct inhibition of bacterial growth by synthetic retinoids [9]. In contrast retinaldehyde (but not Retinoic Acid itself) inhibit Gram positive (but not Gram negative) bacteria in culture [10].

The activities of vitamins A & D have been extensively reported in relation to the host immune response in mycobacterial diseases [4], [8], [11]–[15]. We posit that vitamins will have fundamental and necessary activity in both prokaryotes as well as eukaryotes. We hypothesized that vitamins A and D might directly inhibit prokaryotic growth in general and mycobacterial growth in particular. Any direct inhibitory action of vitamins would be in addition to (and possibly synergistic with) their effect on the immune response of a mycobacterial-infected host [5]–[8].

We herein report on radiometric culture studies of the four fat-soluble vitamins (A, D, E & K) as well at the vitamin A precursor β–carotene and three vitamin A metabolites (retinyl acetate, retinoic acid and 13-cis retinoic acid) on three mycobacterial species. They are the acknowledged human pathogen *M. Tuberculosis (M. tb.)* complex, *M. avium* subspecies *avium* (*M. avium*) pathogenic in immuno-compromised humans and the possibly zoonotic *M. avium* subspecies *paratuberculosis* (MAP) [16].

## Methods

This study was approved by the Research & Development Committee at the VAMC Bronx NY (0720-06-038) and was conducted under the Institutional Radioactive Materials Permit (#31-00636-07).

### Bacterial Culture

Our Bactec® 460 (Becton-Dickinson Franklin Lakes NJ) ^14^C radiometric culture inhibition methods have previously been published in detail [17]–[22]. This system quantifies bacterial growth, or lack thereof, by providing ^14^C in palmitate, an energy source for mycobacterial growth [23]. Vials are assayed on a daily basis, quantifying the amount of ^14^C released as ^14^CO_2_, by the integral detector in the Bactec 460. The data are obtained as a manufacturer determined, arbitrary Growth Units (GU) of 0-999. Because the Bactec 460 is only semi-automatic, and the onerous regulatory requirements of using radionucleotides, this exquisitely sensitive [18] system is being phased out. It is being replaced by the fully automatic, oxygen consumption detecting fluorescent probe MIGT system (Becton-Dickerson NJ.) [24], [25]

The detergent Tween 80 (recommended to minimize mycobacterial clumping [23]) is not used in culture, because of interference with the assay [21], [26]. Strains with the least spontaneous clumping are studied instead. Except for the amount of test agent, every vial has the identical concentration of all constituents (including identical 3.2% concentration of the dissolving agent, DMSO.) In this study, performed in singlicate, eight strains of mycobacteria, four of which are MAP, are evaluated. Two MAP strains had been isolated from humans with Crohn disease “Dominic” (ATCC 43545; Originally isolated by R. Chiodini [27]) and UCF 4 (gift of Saleh Naser, Burnett College of Biomedical Sciences, University of Central Florida, Orlando FL.) [28]. The other two MAP strains were from ruminants with Johne disease, ATCC 19698 and 303 (gift of Michael Collins Madison WI.) The *M. avium* subspecies *avium* strains (hereinafter called *M. avium*) were ATCC 25291 (veterinary source) and *M. avium* 101 (Human isolate from a patient with AIDS; Gift of Clark Inderlied PhD. UC Los Angles CA.) [29]. To study the *M. tuberculosis* complex, we used two BioSafety level 2 strains; Bacillus Calmette Guerin (BCG) *M. bovis* Karlson & Lessel (ATCC 19015) and an avirulent *M. tb* strain; ATCC 25177 (all ATCC from ATCC Rockville MD).

The fat soluble vitamins studied were: vitamin A (Retinol; Axerophthol, -3,7-Dimethyl-9-(2,6,6-trimethyl-1-cyclohexen-1-yl)-2,4,6,8-nonatetraen-1-ol: Sigma Cat # R7632.) The vitamin A precursor studied was β–Carotene (β,β-Carotene, Provitamin A: Sigma Cat # 22040.) We studied three vitamin A metabolites; Retinyl acetate (Retinol acetate, vitamin A acetate: Sigma Cat # R3250) and Retinoic acid (ATRA, Tretinoin, vitamin A acid, all-*trans*-Retinoic acid: Sigma Cat # R2625). Additionally we evaluated 13 cis-Retinoic acid (Isotretinoin, Acutane® Sigma Cat # R3255), a medication used to treat intractable acne and occasionally associated with the manifestation of both Crohn's disease and ulcerative colitis. A commercial source of another structural analog of retinoic acid; 9-cis-Retinoic acid could not be identified.

The other three fat-soluble vitamins studied are: vitamin D (Cholecalciferol; (+)-Vitamin D_3_, 7-Dehydrocholesterol activated, Activated 7-dehydrocholesterol: Sigma Cat # C9576). Vitamin E ((±)-α-Tocopherol DL-all-rac-α-Tocopherol: Sigma Cat # T3251). Vitamin K_1_ (2-Methyl-3-phytyl-1,4-naphthoquinone, 3-Phytylmenadione, Phylloquinone: Sigma Cat # 95271). Our inhibitory antibiotic control is monensin [20] and the non-inhibitory control is the gluterimide antibiotic, phthalimide [21]. (All Sigma, St Louis. MO.) Chemicals are dissolved in DMSO, aliquoted, stored at −80°C, thawed, used once and discarded. Agents are studied at concentrations ranging from 0.1 to 64 µg/ml.

**Figure 1:**
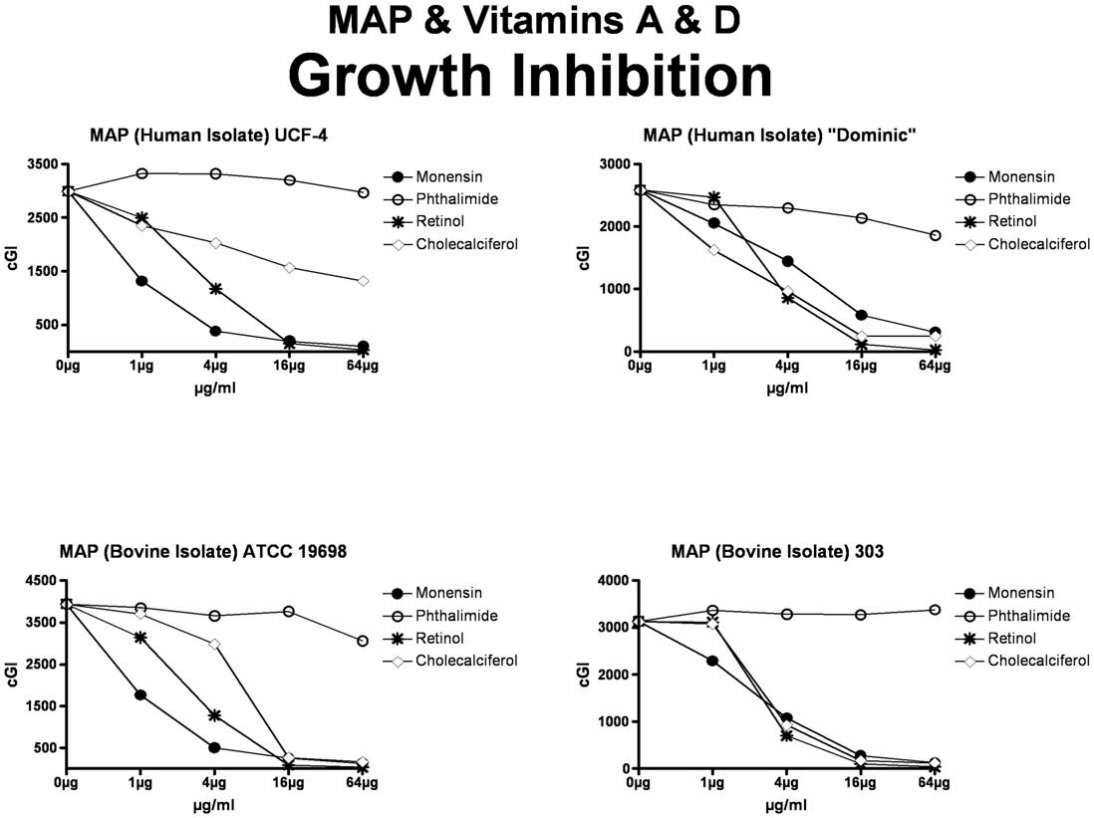
Both vitamins A & D inhibit all four MAP strains studied. Strains from the two upper panels (UCF-4 & Dominic) were isolated from humans with Crohn disease. Strains in the two lower panels were isolated from ruminants with Johne disease. The inhibitory control is Monensin, and the non-inhibitory control is Phthalimide. cGI = cumulative Growth Index.

For clarity and ease of understanding the same data are presented in two ways. For individual mycobacteria we present data from a single experiment graphically (Figures 1–9). These data are presented as the cumulative Growth Index (cGI.) In contrast, for each individual chemical agent studied, data are presented in Tables as the “percent change from control cGI” (Inhibition as “%–ΔcGI”; See [18] for calculation: Tables 1–10).

**Figure 2 pone-0029631-g002:**
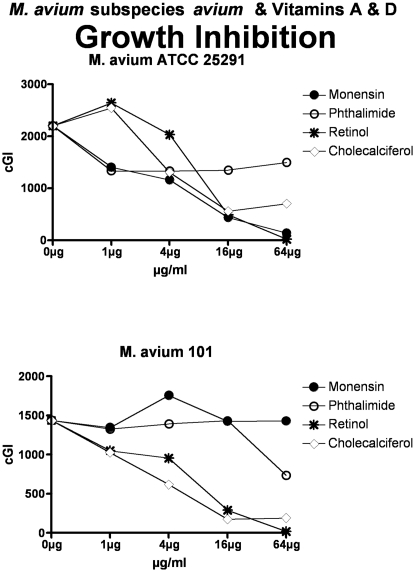
Both vitamins A & D inhibit *M. avium*. The inhibitory control is Monensin, and the non-inhibitory control is Phthalimide. Note that as previously [20], [21], [30], Monensin does not inhibit *M avium* 101. cGI = cumulative Growth Index.

**Figure 3 pone-0029631-g003:**
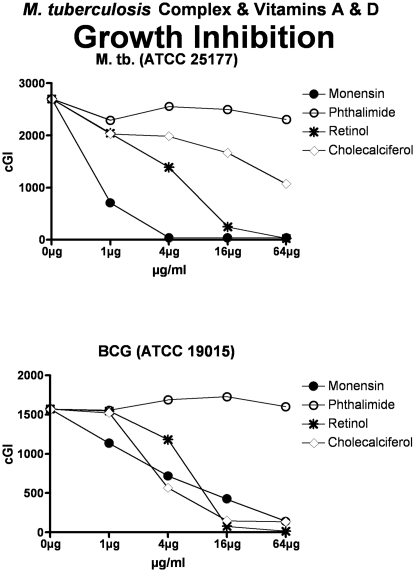
Both vitamins A & D inhibit the *M. tb* complex. Vitamin D is less effective against *M. tb* ATCC 25177. The inhibitory control is Monensin, and the non-inhibitory control is Phthalimide. cGI = cumulative Growth Index.

**Figure 4 pone-0029631-g004:**
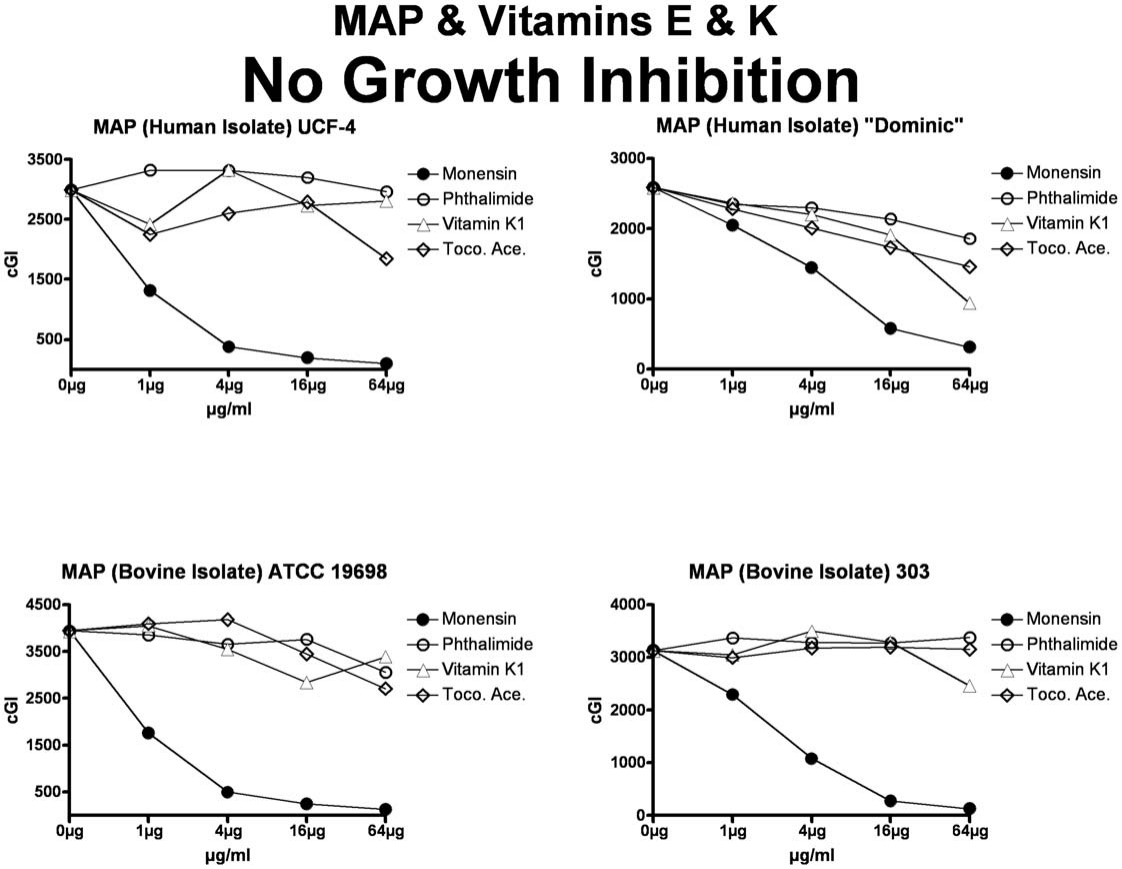
Neither vitamins E nor K inhibit MAP (other than limited inhibition by vitamin E on Dominic.) The inhibitory control is Monensin, and the non-inhibitory control is Phthalimide. cGI = cumulative Growth Index.

**Figure 5 pone-0029631-g005:**
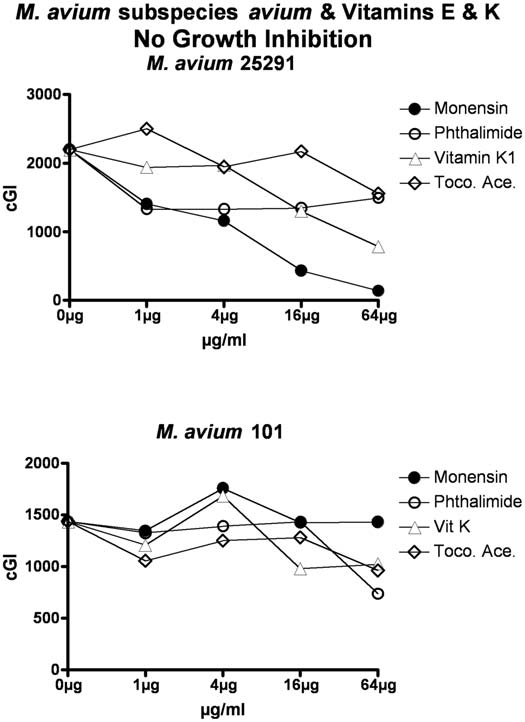
Neither vitamins E nor K inhibit *M. avium*. The inhibitory control is Monensin, and the non-inhibitory control is Phthalimide. Note that as previously [20], [21], [30], Monensin does not inhibit *M avium* 101. cGI = cumulative Growth Index.

**Figure 6 pone-0029631-g006:**
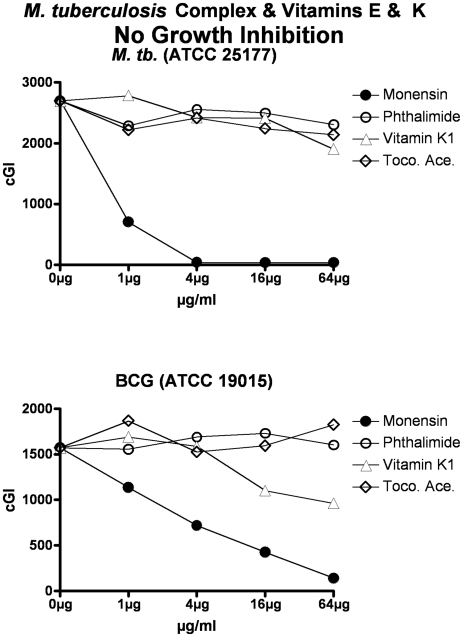
Neither vitamins E nor K inhibit the *M. tb.* complex. The inhibitory control is Monensin, and the non-inhibitory control is Phthalimide. cGI = cumulative Growth Index.

**Figure 7 pone-0029631-g007:**
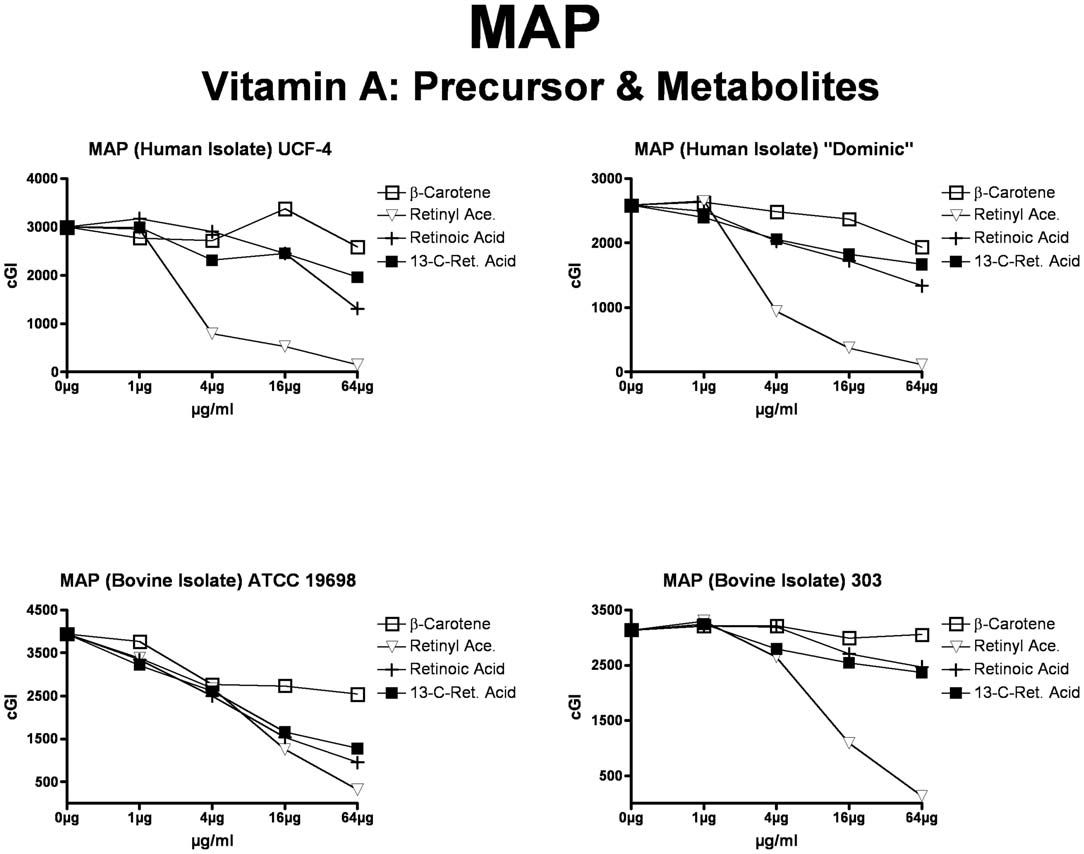
The effects of vitamin A precursors and metabolites on MAP. ß-carotene, the precursor to vitamin A, exhibits no inhibition at the doses studied. Maximal inhibitory activity against all MAP strains is observed with Retinyl acetate (solid black triangles.) Both Retinoic acid and 13-cis Retinoic acid exhibit intermediate inhibition.

**Figure 8 pone-0029631-g008:**
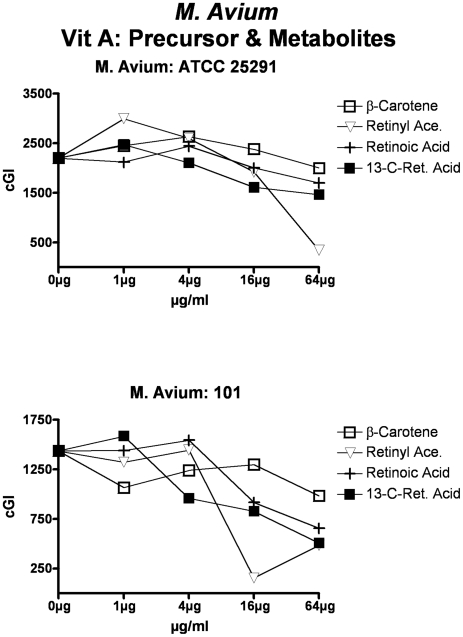
The effects of vitamin A precursors and metabolites on *M. avium*. ß-carotene, the precursor to vitamin A, exhibits no inhibition at the doses studied. Retinyl acetate and 13-cis Retinoic acid have some inhibition.

**Figure 9:**
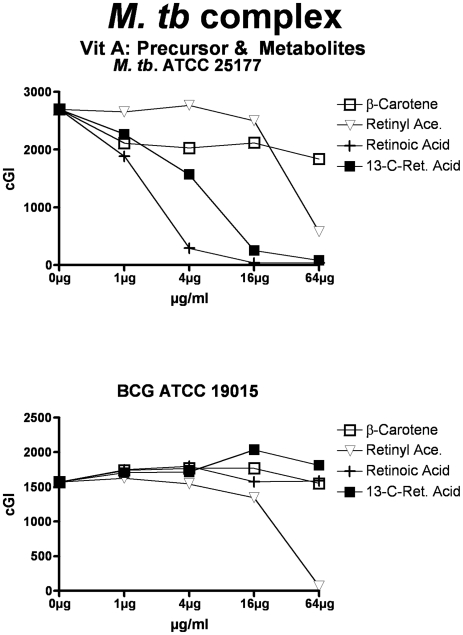
The effects of vitamin A precursors and metabolites on *M. tb. complex.* ß-carotene, the precursor to vitamin A, exhibits no inhibition at the doses studied. Both Retinoic acid and 13-cis Retinoic acid result in dose dependent inhibition on our avirulent strain of *M. tb*. There is no comparable inhibition against Bacillus Calmette-Guerin (BCG).

**Table 1:** Monensin Inhibitory Control.

µg/ml	MAP				M. Avium		*M. tb*. Complex	
	Human Isolate		Bovine Isolate				M. tb.	BCG
	UCF-4	Dominic	19698	303	25291	101	25177	19015
1	−56%	−21%	−55%	−27%	−36%	−6%	−74%	−28%
4	−87%	−44%	−87%	−66%	−47%	22%	−98%	−54%
16	−94%	−78%	−94%	−91%	−80%	−1%	−99%	−73%
64	−97%	−88%	−97%	−96%	−94%	0%	−99%	−91%

**Table 2 pone-0029631-t002:** Phthalimide Non-Inhibitory Control.

µg/ml	MAP				M. Avium		*M. tb*. Complex	
	Human Isolate		Bovine Isolate				M. tb.	BCG
	UCF-4	Dominic	19698	303	25291	101	25177	19015
1	11%	−9%	−2%	7%	−39%	−8%	−15%	−1%
4	11%	−11%	−7%	5%	−40%	−3%	−5%	7%
16	7%	−17%	−5%	4%	−39%	0%	−7%	10%
64	−1%	−28%	−22%	8%	−32%	−49%	−15%	2%

**Table 3 pone-0029631-t003:** Vitamin A: retinol.

µg/ml	MAP				M. Avium		*M. tb*. Complex	
	Human Isolate		Bovine Isolate				M. tb.	BCG
	UCF-4	Dominic	19698	303	25291	101	25177	19015
1	−17%	−5%	−20%	−1%	20%	−27%	−24%	−2%
4	−61%	−67%	−68%	−77%	−8%	−34%	−48%	−25%
16	−95%	−96%	−98%	−97%	−78%	−80%	−91%	−95%
64	−99%	−99%	−99%	−99%	−99%	−99%	−99%	−99%

**Table 4 pone-0029631-t004:** Vitamin D Cholecalciferol.

µg/ml	MAP				M. Avium		*M. tb*. Complex	
	Human Isolate		Bovine Isolate				M. tb.	BCG
	UCF-4	Dominic	19698	303	25291	101	25177	19015
1	−22%	−37%	−6%	−2%	15%	−29%	−25%	−3%
4	−32%	−63%	−24%	−71%	−41%	−57%	−26%	−64%
16	−48%	−91%	−93%	−94%	−75%	−88%	−38%	−91%
64	−56%	−90%	−96%	−96%	−68%	−87%	−60%	−91%

**Table 5 pone-0029631-t005:** Vitamin E DL-α-Tocopherol Acetate.

µg/ml	MAP				M. Avium		*M. tb*. Complex	
	Human Isolate		Bovine Isolate				M. tb.	BCG
	UCF-4	Dominic	19698	303	25291	101	25177	19015
1	−25%	−12%	4%	−5%	14%	−27%	−18%	19%
4	−13%	−22%	6%	1%	−12%	−13%	−10%	−3%
16	−7%	−33%	−12%	2%	−1%	−11%	−17%	1%
64	−38%	−44%	−31%	1%	−29%	−33%	−21%	16%

**Table 6 pone-0029631-t006:** Vitamin K1.

µg/ml	MAP				M. Avium		*M. tb*. Complex	
	Human Isolate		Bovine Isolate				M. tb.	BCG
	UCF-4	Dominic	19698	303	25291	101	25177	19015
1	−19%	−9%	3%	−3%	−12%	−16%	3%	8%
4	11%	−15%	−10%	12%	−10%	17%	−10%	1%
16	−9%	−26%	−28%	5%	−41%	−32%	−11%	−30%
64	−6%	−64%	−14%	−21%	−64%	−29%	−29%	−39%

**Table 7 pone-0029631-t007:** β-Carotene.

µg/ml	MAP				M. Avium		*M. tb*. Complex	
	Human Isolate		Bovine Isolate				M. tb.	BCG
	UCF-4	Dominic	19698	303	25291	101	25177	19015
1	−8%	2%	−4%	2%	11%	−26%	−22%	11%
4	−9%	−4%	−30%	2%	20%	−14%	−25%	13%
16	13%	−8%	−31%	−5%	8%	−10%	−21%	13%
64	−14%	−25%	−36%	−3%	−9%	−32%	−32%	−1%

**Table 8 pone-0029631-t008:** Retinyl Acetate.

µg/ml	MAP				M. Avium		*M. tb*. Complex	
	Human Isolate		Bovine Isolate				M. tb.	BCG
	UCF-4	Dominic	19698	303	25291	101	25177	19015
1	−1%	2%	−14%	5%	36%	−8%	−2%	3%
4	−74%	−64%	−32%	−16%	18%	1%	3%	−2%
16	−82%	−86%	−68%	−65%	−13%	−89%	−7%	−14%
64	−95%	−96%	−92%	−96%	−84%	−66%	−78%	−96%

**Table 9 pone-0029631-t009:** Retinoic Acid.

µg/ml	MAP				M. Avium		*M. tb*. Complex	
	Human Isolate		Bovine Isolate				M. tb.	BCG
	UCF-4	Dominic	19698	303	25291	101	25177	19015
1	6%	−4%	−15%	2%	−4%	0%	−30%	11%
4	−3%	−22%	−37%	2%	11%	7%	−89%	15%
16	−18%	−33%	−61%	−14%	−9%	−36%	−99%	0%
64	−56%	−48%	−76%	−21%	−23%	−54%	−99%	1%

**Table 10:** 13 Cis-Retinoic Acid.

µg/ml	MAP				M. Avium		*M. tb*. Complex	
	Human Isolate		Bovine Isolate				M. tb.	BCG
	UCF-4	Dominic	19698	303	25291	101	25177	19015
1	0%	−7%	−18%	−21%	12%	10%	−16%	9%
4	−23%	−20%	−34%	−20%	−5%	−33%	−42%	9%
16	−18%	−29%	−58%	−34%	−27%	−42%	−91%	30%
32	−35%	−35%	−68%	−44%	−34%	−65%	−97%	15%

For simplicity and comprehensibility the data in each of Figues 1–6 are for only two of the four agents tested. For ease of comparison the inhibitory (Monensin) and non-inhibitory control (Phthalimide) are repetitively presented. Data for vitamins A & D are presented in Figure 1 (MAP), Figure 2 (*M. avium*) & Figure 3 (*M. tb.* complex) and vitamins E & K are presented in Figure 4 (MAP), Figure 5 (*M. avium*) & Figure 6 (*M. tb.* complex.) The vitamin A precursor and structural analogs are presented in tabular form. (β-Carotene; Table 7: Retinol acetate; Table 8: Retinoic acid; Table 9: and 13-cis Retinoic acid; Table 10.) Data for vitamin A precursors and analogs on mycobacterial species and subspecies are presented as Figures (MAP = Figure 7: *M. avium* = Figure 8 & *M. tb.* complex = Figure 9.).

## Results

In this study we show that all MAP and both *M. tb* complex strains are inhibited by Monensin (Table 1 and Figures 1 & 4 & Table 1 & Figures 3 & 6.) This corroborates our previous findings with Monensin [20], [21], [30]. As previously [20], [21], [30], Monensin does not inhibit one of our two *M. avium* control strains (*M. avium* 101: Table 1 and Figures 2 & 5), attesting to the reliability and reproducibility of our assay.

The non-inhibitory control that we use is Phthalimide, a gluterimide antibiotic that has no mycobacterial inhibition [21]. In this study, as previously [21], [30], [31], Phthalimide has no dose-dependent inhibition against any of the mycobacterial strains tested (Table 2 and Figures 1–6.)

Vitamin A causes dose dependent inhibition of all MAP, *M. avium* and *M. tb* complex strains studied (Table 3 & Figures 1, 2 & 3) with 98%-ΔcGI at 16 µg/ml for MAP ATCC 19698. The precursor to vitamin A, β-Carotene has no dose dependent inhibition on six of eight mycobacterial strains and negligible inhibition on two MAP strains (Table 7 & Figures 7, 8 & 9.) In contrast, the three vitamin A metabolites (Figures 7, 8 & 9); retinyl acetate (Table 8), retinoic acid (Table 9), and 13-cis retinoic acid (Table 10) result in dose dependent inhibition of all three species studied, but with intriguing interspecies variations. Retinyl Acetate is most active against MAP (Figure 7 & Table 8; Dominic 96%-ΔcGI at 64 µg/ml.) Retinoic acid and 13-cis retinoic acid are most active against *M. tb* (98%-ΔcGI at 16 µg/ml), but have no inhibition against BCG (Figure 9 & Tables 9 & 10.) *M. avium* is the least susceptible to these vitamin A metabolites (Figure 8 & Tables 8–10).

Vitamin D causes dose dependent inhibition of all MAP strains studied (Table 3 & Figure 1). However, vitamin D is not as potent an inhibitor against the two MAP human isolates (UCF-4; 56%-ΔcGI at 64 µg/ml and Dominic) as it is on the two MAP bovine isolates (Table 4 and Figure 1.) Likewise, vitamin D inhibits all *M. avium* (Table 4 & Figure 2) and *M. tb.* complex (Table 4 & Figure 3) strains studied. Finally, vitamin D is less effective than vitamin A against all four *M. avium* and *M. tb* complex strains studied (Tables 3 & 4 and Figures 1–3.).

In contrast, vitamin E (Table 5; Figures 4, 5, & 6) results in inhibition of only one of the eight mycobacterial strains studied, MAP Dominic (Table 5 & Figure 4.) Even then, the maximal inhibition of vitamin E on Dominic (44%-ΔcGI at 64 µg/ml; (Table 5 & Figure 4) is far less than is observed with either vitamin A or D.

Vitamin K has no effect on the growth on any of the three mycobacterial species studied (Table 6; Figures 4, 5, & 6.).

## Discussion

To our knowledge this is the first study showing dose-dependent inhibition, in radiometric culture, of three mycobacterial species (the *M. tb.* complex, *M. avium* and MAP) by two of four fat-soluble vitamins; vitamins A & D. In contrast, vitamin K has no, and vitamin E negligible effect. These therefore provide appropriate non-inhibitory experimental controls. Our observations cannot be ascribed to the acidic nature of vitamin A or its analogs as the pH remains within the manufacturer's recommended range of pH 6.6±2 in the final 5 ml incubation volume (data not presented.) The mechanism(s) by which vitamins A & D inhibit mycobacterial growth, and whether they have similar inhibition on virulent and/or multi-drug resistant *M. tb.*, remains to be determined.

Our finding are directly contradictory to those of Flemetakis *et. al.* who concluded that there was no direct retinoid effect on bacteria *in vivo* [9]. Others find that vitamin A and retinoic acid have no antibacterial activity, whereas retinaldehyde does [10]. Neither study evaluated mycobacteria in radiometric culture. We, and others [32], conclude that when evaluating mycobacterial growth kinetics, liquid radiometric [23] data provide exquisitely sensitive data of bacteriostatic in addition to bactericidal effects.

Inhibition of mycobacterial growth by vitamins A & D has been ascribed to down regulation of the tryptophan-aspartate-containing coat protein (TACO) gene in the human macrophage [8], [33]. Our data are compatible with an additional hypothesis. It is that vitamins D, A and vitamin A metabolites have a heretofore unproven, independent and probable synergistic antimycobacterial inhibitory action that complements the immune response of multicellular organisms.

The vitamin A precursor, β-Carotene, does not inhibit mycobacterial growth. This indicates that mycobacterial mechanisms to convert β-Carotene to vitamin A are inadequate to produce sufficient vitamin A levels to inhibit mycobacterial growth. We conclude that the subspecies specific, idiosyncratic, inhibition of the three vitamin A metabolites merit further study, as do structural analogs of vitamin D.

We posit that multiple agents have underappreciated activity against prokaryotes in addition to well-documented eukaryotic activity. For example, we [17], [18], [20]–[22], [30], [31], and others [34], [35], have shown inhibition of MAP growth with medications used to treat “autoimmune” and “inflammatory” diseases. In the present study we show direct inhibition of mycobacterial growth by vitamins A and D in culture. We conclude that the scientific community has neglected the potential direct prokaryotic effects of vitamins, emphasizing instead the indirect role that vitamins have in enhancing the immune response of an infected host.

Our radiometric assay [23] is sufficiently sensitive to identify mycobacterial growth enhancement in culture [31]. Using it, we have corroborated the classic study of Bernheim in 1940 [36] showing that salicylic acid increased oxygen consumption by the tuberculosis bacillus. Additionally, we showed growth enhancement of mycobacteria by vitamin B3 (nicotinamide), nicotinic acid (a tobacco constituent) and α &ß NAD [31]. In 1940 the possibility that vitamin K enhanced the growth of MAP was considered [37]. (see [38] for review). The identification of the necessary, and potent, iron chelating mycobactins of *M. phlei* [39], [40] (see [41] for review), left unresolved a possible enhancing role of vitamin K on MAP growth [37]. In this present study we observe no growth enhancement by vitamin K_1_. It is of interest however, that vitamin K_2_ (menaquinone), which we did not evaluate, may inhibit mycobacterial growth [42]. We now conclude that vitamin K_1_ has no effect on the growth of three mycobacterial species, including MAP.

This study does not address how vitamin concentrations that are inhibitory in our culture system, relate to concentrations actually found in multicellular organisms. For example our “normal” laboratory range in humans for circulating vitamin A is 0.3–0.9 µg/ml, a level below those tested in our studies (1–64 µg/ml.) Lipophylic antibiotics, such as azithromycin, may achieve tissue levels 1,000 fold greater than circulating values [43]. Since “normal” laboratory concentrations are “circulating” plasma levels, they may vastly underestimate concentrations that these fat-soluble vitamins achieve in lipid rich regions, such as prokaryotic and eukaryotic cell walls and other lipophylic regions within cells.

Prevailing dogma considers that all of the anti-mycobacterial activity of vitamins A & D is mediated, indirectly, via enhancement of the immune system of the eukaryotic host. Our data are compatible with an alternative hypothesis: In addition to their eukaryotic activity, vitamins A & D may directly inhibit mycobacteria within the eukaryotic host. Similarly whether vitamins may act as naturally occurring “antibiotics” and help prevent a host infected by mycobacteria from progressing to active disease will require extensive and complicated, IRB compliant, additional studies. Nevertheless, it is of considerable interest that low exposure to sunlight, which is associated with diminished vitamin D levels [44], is associated with an increase in the incidence of Crohn disease [45], which may be caused by MAP [46].
